# Providing Healthy Work Environments Through Facilitation of Nurse Educators’ Self-Leadership in Academic Settings: A Qualitative Explorative Study

**DOI:** 10.1177/23779608251366955

**Published:** 2025-08-06

**Authors:** Gisela H van Rensburg, Vhothusa Edward Matahela, Sara Horton-Deutsch

**Affiliations:** 1 Department of Health Studies, 56866 University of South Africa, Pretoria, South Africa; 2 16152 University of SanFrancisco School of Nursing and Health Professions, CA, USA

**Keywords:** educational institution, healthy work environments, nurse educators, self-leadership, taking own initiative

## Abstract

**Introduction:**

In today's dynamic academic landscape, self-leadership is an essential attribute for employees, including nurse educators. Various factors within educational institutions can influence the ability of nurse educators to exercise self-leadership effectively, which in turn impacts their performance and workplace well-being.

**Objective:**

This study explored nurse educators’ understanding of self-leadership and examined their perspectives on how fostering self-leadership can contribute to creating a healthier academic work environment.

**Methods:**

A qualitative, exploratory, and descriptive research design was employed. Focus group interviews were conducted with nurse educators from four educational institutions in South Africa, and the data were analyzed using Tesch's qualitative content analysis.

**Results:**

The study identified two key themes: (1) *Empowering Nurse Educators to Take Initiative*, which includes four subthemes, and (2) *Creating a Supportive and Healthy Academic Work Environment,* also with four subthemes.

**Conclusion:**

Academic institutions that collaborate with nurse educators and actively support their self-leadership initiatives play a critical role in fostering healthy and productive work environments.

## Introduction

South African educational institutions are implementing higher education reforms, including the transitioning of all nursing qualifications to align with the higher education band and international standards to improve the quality of healthcare services (Crowley & Daniels, 2023; Matahela & Makhanya, 2024). As these institutions implement the reforms, nurse educators are once more called upon to provide leadership and guidance in a plethora of tasks. In addition, nurse educators need to adapt to change with ease and commit to being lifelong learners with a desire to learn and acquire new skills so that they keep abreast with industry trends (Fateh et al., 2021).

Teaching nurses is naturally interesting. Clark (2025) states that environmental (organizational) factors may lead to a waning of nurse educators’ enthusiasm to teach. These factors are mainly related to the widely reported reduction of government budget toward nurse training in the country (Schütz, 2021). The factors include inadequate teaching and learning infrastructure, unavailability of mentors, lack of investment in nurse educators’ professional development, and lack of collaboration (Bvumbwe & Mtshali, 2018). This has led to some nurse educators describing their working environment as unappreciative and characterized by unsupportive leaders, lack of meaningful acknowledgment, lack of teamwork, resistance to change, and shortage of equipment (Matahela, 2021).

The decreasing engagement and motivation of nurse educators in teaching have been further exacerbated by adverse effects on their sense of self (Matahela, 2021). Studies by Christensen et al. (2021) and Clark (2025) report a worrying trend of uncivil behaviors perpetrated by nursing students toward nurse educators. Amongst these disheartening and adverse effects on motivation are the use of discourteous language, belittling remarks, and disregard toward nurse educators, which persist and continue due to the sharing of these matters on social media networks (Christensen et al., 2021). Oftentimes, the institutions fail to act against the perpetrators of discourteous acts, further exacerbating perceptions of betrayal by the institution (Solheim, 2024).

Challenging encounters during teaching and learning require 21st-century approaches: they need self-leading educators who are innovative and resilient. Self-leading individuals are internally motivated and have the determination and self-confidence to succeed and persist through adversities until goal achievement (Fateh et al., 2021). However, the self-leadership practices are amenable to work environments that promote innovation and creativity amongst workers (Fateh et al. 2021). Such a supportive work environment would be appropriate so that nurse educators can provide a reciprocal environment for students (Kaylor & Johnson, 2019).

Nurse educator self-leadership became particularly significant as academic settings adopted remote and flexible working conditions (Sjöblom et al., 2022). Even postpandemic, nurse educators continue to exhibit more self-directed work behaviors and are expected to take greater responsibility for their teaching tasks, rather than relying solely on designated leaders. It is therefore essential to explore how facilitating nurse educators’ self-leadership in healthy academic work environments can help them effectively adapt to these evolving expectations.

## Literature Review

Self-leadership is a comparatively different view of leadership that proposes that employees have the capacity to lead themselves without external influence (Angus et al., 2024). Self-leadership is defined as a process by which individuals can influence themselves to an extent that they can self-direct and self-motivate to perform optimally. This can be achieved through setting their own standards and objectives, and intentionally analyzing their implemented activities (Goldsby et al., 2020; Pearce & Manz, 2020).

The theoretical framework that guided this qualitative study is Manz's (1986) *Expanded theory of self-influence processes in organisations*. The basis of this theoretical foundation is that organizational members are in possession of individual intrinsic mechanisms for self-control, which are applied alongside the organization's own performance measurement and reward systems. The self-leadership theory suggests that there are three self-leadership strategies that are intended to derive positive self-influence that leads to individual effectiveness: behavior-focused strategies, natural reward strategies, and constructive thought pattern strategies (Pearce & Manz, 2020).

Behavior-focused self-leadership strategies are designed to increase self-awareness so that an individual can manage his or her behavior to achieve the required outcome, particularly those that are essential but deemed unpleasant tasks (Knotts et al., 2022). Natural reward strategies are intended to nurture circumstances that intrinsically motivate or reward an individual by virtue of them being pleasant to partake in (Neck & Houghton, 2006). Finally, constructive thought pattern strategies are designed to facilitate and develop a positive mental image of the task to bring about an improved performance (Neck & Houghton, 2006). Constructive thought pattern strategies comprise identifying defective beliefs and assumptions and replacing them with constructive and habitual ways of thinking as well as positive self-talk (Pearce & Manz, 2020). Owing to their possession of and effective use of self-leadership strategies, self-leading individuals are well-resourced to achieve the set goals (Inam et al., 2021).

Self-leadership is an internal process, and as such, external factors such as the work environment (organizational climate) and institutional managers and colleagues (social climate) play a crucial role in an individual's implementation of self-leadership strategies (Harari et al., 2021). Nurse educators do not exist in isolation but rely on effective interpersonal relationships with peers to succeed. Peer support affords nurse educators opportunities to enhance teamwork to improve the quality of educational provisions in the academic setting (Woods et al., 2022). Keddie et al. (2023) reiterate that educators yearn for a work environment that is perceived to facilitate collegial respect, a bureaucracy-free environment, and provision of autonomy, competence, and relatedness. Academic working environments that support educators’ autonomy, competence, and relatedness promote their intrinsic motivation to teach (Santana-Monagas et al., 2022). A healthy academic environment can enhance staff motivation and fulfillment. However, each nurse educator is responsible for staying relevant and thriving within the institution. This includes setting goals, maintaining self-awareness, staying motivated, and monitoring progress—key aspects of self-leadership (Paulino, 2023). Most research on self-leadership in education focuses on internal factors related to goal achievement (George-Puskar et al., 2024). This study examines how external factors, such as a supportive work environment, can foster self-leadership in nursing educators. It also explores their understanding of self-leadership within an academic setting.

Some scholars have lamented the dearth of research on the self-leadership concept and its practices for nurse educators in educational settings (Matahela & Van Rensburg, 2021, 2023a; Nowell et al., 2017). In addition, it is purported that leaders in many organizations cannot discern leading in a way that promotes their followers’ self-leadership (Ntshingila et al., 2021). Given the nurse educators’ working context of an environment characterized by constant changes, and the potential benefits attained from engaging in self-leadership practices, the following research question arose: *How can nurse educators’ self-leadership be facilitated to ensure a healthy work environment?* This article aims to explore and describe the educators’ understanding of nurse educator self-leadership and its facilitation to create a healthy work environment within their work environment.

## Materials and Methods

### Design

This study used an exploratory, descriptive qualitative design to gain an in-depth understanding of the phenomenon. This approach was chosen to explore nurse educators’ lived experiences, perceptions, and the meanings they assign to self-leadership. Given the complexity of self-leadership in nursing education, a qualitative design allowed for rich, nuanced insights that quantitative methods might not capture. This method is especially effective for understudied topics (Hunter et al., 2019).

### Participants and Inclusion Criteria

A sampling strategy of purposive sampling was employed with the rationale that this approach would help identify individuals who could provide contextual meaning of the phenomenon. Nurse educators involved in teaching and learning activities at purposively selected private and public nursing education institutions in two South African provinces formed the population of the study. These provinces were chosen not only for their similar governance and developmental profiles but also because they had an almost equal representation of diverse types of nursing education institutions, including private and public nursing colleges as well as universities. Additionally, these provinces had historically produced the highest number of nurse graduates over time.

Potential and willing participants who met the selection criteria were approached beforehand through research coordinators in the educational institutions. To be included in the study, participants had to be involved in teaching and learning tasks on a permanent employment basis for at least a year-long period and were readily available to participate in the study. The authors regarded a year's experience as adequate time for an individual to have developed a perception of their self-leadership in a work setting, thus participating in this study.

### Participant Characteristics

Participants’ sociodemographic data are displayed in Table 1.

**Table 1.** Sociodemographic Characteristics of Study Participants.

Characteristic	Details
Gender	All female (26), no males
Age	Between 25 and 60 years
Ethnicity	African: 17; Colored: 6; White: 3
Educational Attainment	Doctorate: 3; Master's: 10; Postgraduate Diploma: 6; Bachelor's Degree: 7
Clinical Nursing Experience	3 to 16 years
Nursing Education Experience	1 to 21 years
Type of Nursing Education Institution	Private Nursing School: 12; Public Nursing College: 8; University Department: 6
Job Title at Institution	Senior Lecturer: 11; Lecturer: 10; Clinical Lecturer: 3; Contract Lecturer: 2
Responsibilities at Institution	Teaching theory, clinical instruction, research supervision, quality assurance, student advising, and administrative tasks

All the 26 participants who participated in the study were female, with no male participants. In South Africa, male nurses mainly work in nursing management, occupational health, and critical care environments. It is a common phenomenon that nursing education institutions may have no male nurse educators. Although male nurses qualify as nurse educators and register with the regulatory body as such, they prefer not to take on educational roles. No participant withdrew from the study.

### Data Collection

The original data for this study were collected in 2018 and 2019 through focus group interviews with nurse educators. To enhance its applicability to contemporary discussions on healthy work environments, the data were revisited and refined, aligning with emerging insights in the field. Additionally, an updated literature review was conducted to validate the relevance of the findings within the current academic and practical context, ensuring they reflect the latest developments and perspectives in the discipline. The interviews were conducted in English by the second author, a male researcher with extensive experience in nursing education leadership. At the time of the study, the second author was employed as a manager at a different organization, bringing an external yet informed perspective to the research process. He held a Master's degree in Nursing Education and had significant expertise in qualitative research methodologies. His leadership background in nursing education and qualitative inquiry enhanced the depth of discussions, ensuring a rigorous and contextually grounded data collection process**.**

Four focus groups (FG1 to FG4) were conducted, each comprising between five and eight nurse educators (FG1: 7; FG2: 5; FG3: 6; FG4: 8), with interviews held in the participants’ natural academic settings to encourage authentic engagement and contextually grounded insights. No nonparticipants were present during the interviews, which were conducted in private spaces to ensure confidentiality, minimize external influence, and create a conducive environment for open and honest discussions facilitated solely by the second author. Ethical clearance was obtained from the University's Research and Ethics Committee (Ethical Clearance Certificate: REC-012714-039), as well as from the research and ethics structures of the participating institutions. Informed consent was secured from all participants. Each interview lasted approximately 55 min, and data saturation was reached by the fourth focus group.

The central questions that guided the interviews were:

- What is your understanding of nurse educator self-leadership within an educational institution setting?
- In your opinion, how can the educators’ self-leadership be facilitated to ensure a healthy work environment within the educational institution?

An interview guide was developed based on key self-leadership concepts rather than a rigid set of predetermined questions. This approach allowed for flexibility in responses, fostering deeper exploration of both individual and institutional factors that influence self-leadership. Participants received the interview guide in advance, enabling them to reflect on their experiences beforehand and engage in more meaningful discussions. The interview process was dynamic, with probing guided by participants’ responses, ensuring that emerging themes aligned with self-leadership strategies and the broader organizational climate in which nurse educators operate.

To ensure the relevance of our findings within the evolving academic landscape, the researchers conducted a secondary data analysis in 2023. This analysis integrated insights from recent institutional developments, policy changes, and contemporary discourse in nursing education. Additionally, a comprehensive literature review was updated through 2024 to strengthen the study's foundation, ensuring that the findings remain grounded in current scholarship and practical applications.

### Data Analysis

This study adheres to the Consolidated Criteria for Reporting Qualitative Research (COREQ) guidelines (Tong et al., 2012) to ensure comprehensive reporting. Transcripts and field notes were independently analyzed by two of the authors, as well as by an independent co-coder who is a doctoral-trained nurse with expertise in qualitative research methods, following Tesch's protocol of qualitative content analysis (Creswell & Creswell, 2023). The analysis was informed by Manz's (1986) theoretical framework, which offered a structured approach to interpreting self-leadership strategies within the participants’ narratives. Specifically, the data were examined to identify instances where nurse educators applied behavior-focused strategies, leveraged natural rewards, and engaged in constructive thought patterns. Additionally, the study explored how institutional and social environments either supported or impeded these self-leadership practices.

Field notes formed an integral part of the data collection process as they were used to capture nonverbal cues and nuances that would not be evident in the audio recordings alone. Field notes were taken simultaneously with the focus group interviews, allowing for the immediate capturing of nonverbal reactions, body language, and the general atmosphere in the room in which the interview took place, which provided a richer context to the verbal responses of the participants. Additional notes were made after the interviews to reflect on any significant observations or themes that emerged. Taking field notes after the interview allowed the researcher to document initial impressions and thoughts that might inform subsequent analysis. Therefore, the field notes added depth to the data, allowing for a more nuanced analysis of the participants’ perceptions regarding self-leadership and how self-leadership can be facilitated to ensure a healthy work environment.

The analysis entailed: careful reading of all the transcripts and noting down ideas, making a list of all topics and clustering similar ones together, and arranging the topics into major topics, unique topics, and leftovers. Thereafter, followed by coding, which was written next to appropriate segments of text, and the most descriptive wording for topics arranged as categories. Interrelationships between categories were depicted, which informed a final decision to abbreviate each category and write codes using alphabets. Data analysis was conducted manually without the use of software, which allowed for a more immersive process, enabling the researchers to deeply engage with the context and meaning of participants’ responses.

A consensus meeting was conducted with the independent coder to review and discuss the separately identified themes and subthemes. During this process, themes were critically examined to ensure their alignment with self-leadership constructs, particularly in how participants expressed their ability to regulate their behaviors (behavior-focused strategies), cultivate intrinsic motivation (natural reward strategies), and reframe challenges positively (constructive thought pattern strategies; Pearce & Manz, 2020).

The discussions led to an agreement on the relationships between the themes and subthemes, which were systematically arranged in columns to provide a clear and structured representation of these connections. Additionally, the descriptive labels assigned to themes and subthemes were reassessed to ensure they accurately reflected participants’ experiences while remaining theoretically grounded in self-leadership principles (Nowell et al., 2017). The audiotape, transcripts were printed for analysis, and field notes were then preserved under lock and key.

### Ethical Considerations

The study adhered to the ethical principles of the 2013 revision of the Helsinki Declaration (initially established in 1975). Participants provided informed consent after receiving both oral and written explanations about the voluntary nature of the focus group interviews and their right to withdraw at any time without explanation. Confidentiality was maintained by encouraging participants to avoid identifying themselves or others during interviews. The University's Research and Ethics Committee and the Research Setting involved approved the study. Informed consent forms were signed after participants’ questions and concerns were addressed and were securely stored to ensure confidentiality.

### Trustworthiness

Lincoln and Guba's (1985) strategies were used to ensure trustworthiness of the qualitative research, namely, credibility, transferability, dependability, and confirmability. Credibility was ensured by presenting data on the self-leadership of nurse educators in an accurate way, so that other nurse educators who share that experience would immediately recognize the descriptions. This was achieved through prolonged engagement, triangulation, member-checking, and persistent observation. Transferability was enhanced by providing a comprehensive description of the research methodology, enabling readers to assess the applicability of findings to other contexts (Blanche et al., 2022). This detailed account includes the research design, data collection methods, and analysis processes.

The study explores and describes nurse educator self-leadership and strategies to facilitate a healthy work environment using purposively selected participants. Verbatim statements from these information-rich participants were provided. Additionally, the findings were supported by relevant existing literature, increasing transferability by grounding the conclusions in established research. Dependability was maintained by providing a comprehensive description of the research methodology, while confirmability was ensured by means of an audit trail, triangulation, and consensus discussions between the second author and the independent coder.

Reflexivity was an integral part of this study and was used to enhance trustworthiness by acknowledging and mitigating potential researcher biases. As nurse educators and qualitative researchers, the research team was aware of the potential for preexisting beliefs and professional experiences to influence the interpretation of data related to self-leadership in nursing education.

To maintain analytical neutrality, the following strategies were employed:

- Documentation of assumptions: Before and during the research process, the team recorded personal beliefs and potential biases related to self-leadership to foster self-awareness and transparency.
- Peer debriefing: Regular discussions were held with research peers not involved in the study to challenge interpretations and promote critical reflection.
- Independent co-coding: An external qualitative researcher was involved in coding and theme development to validate the analytic process. Consensus meetings were held to discuss discrepancies and ensure that the emerging themes authentically represented participants’ perspectives.

## Results

The participants’ understanding of nurse educator self-leadership and how this phenomenon can be facilitated to ensure a healthy work environment in an educational institution is presented in the form of two themes: *self-leadership means nurse educators being supported to take their own initiatives*, with its four subthemes, and *a supportive healthy academic work environment*, with its four subthemes, as depicted in Table 2. Each focus group of nurse educators was interviewed separately at different times rather than all at once. Participants were identified using an acronym (e.g., F1G1, where F1 denotes Female Participant 1 in the first focus group interview) to ensure clarity in reporting responses. The selection of focus groups followed a structured approach to capture diverse perspectives while maintaining coherence within each group.

**Table 2.** Themes and Subthemes.

Themes	Subthemes
Self-leadership means nurse educators being supported to take own initiatives	Setting goals for the self toward a clear vision
	Taking ownership in self-reflection on own behavior
	Serving as role models through self-care
	Self-development
A supportive healthy academic work environment	Mentoring
	Collaborating
	Meaningful recognition
	Supportive management leadership style

The results represent the participants’ shared understanding of self-leadership and how it can be facilitated in an educational setting. Since data were collected through focus group interviews, it is recognized that group dynamics may have shaped participants’ responses. Some individuals might have aligned with dominant opinions or hesitated to express dissenting views, potentially affecting the breadth of perspectives shared. To mitigate these effects, the researchers incorporated strategies to encourage open dialog, such as emphasizing confidentiality, fostering a nonjudgmental environment, and using skilled facilitation techniques to ensure diverse viewpoints were heard. Additionally, probing questions were used to elicit individual reflections beyond group consensus. Triangulation with secondary data sources, such as literature and institutional documents, further strengthened the validity of the findings and helped contextualize participant responses.

### Theme 1: Self-Leadership Means Nurse Educators Being Supported to Take Own Initiatives

In this theme, participants understood self-leadership as taking own initiatives aimed at improving teaching and learning processes, with institutional support. The participants needed support in realizing their set goals, self-reflection, serving as role models, and self-development.

#### Subtheme: Setting Goals for the Self Toward a Clear Vision

The subtheme “setting goals for the self towards a clear vision” deals with nurse educators practicing self-leadership through self-goal setting as part of their vision. The participants viewed their self-leadership as committing thoughts and behaviors in the development of clearly set out personal and professional goals and laying out achievable action plans. For example, a participant expressed the following statement relative to goal setting:

If you don’t have goals, you don’t have direction. As a novice educator my short-term goal is to develop confidence in facilitating lessons, work on my communication and interpersonal skills, set up assessments et cetera. My long-term goal is to enroll for a PhD. (G1 P4)

The participants viewed their self-leadership as formulating a personal vision that articulates their quest for a teaching career and beyond teaching. The following quote illustrates their viewpoint:

I believe as a nurse educator I must have a vision for my professional career, I must know where it is leading me, I must draw up a plan to describe how I will reach there. (G1 P1)

The participants believed that identifying and setting goals was a self-leadership practice, as this action would assist them in regulating their behaviors and monitoring their teaching performance. In addition, they considered their self-leadership as formulating a vision that intentionally makes a pronouncement about their teaching career and other activities outside of teaching. Thus, nurse educators should set realistic goals, have positive mindsets, identify sustainable initiatives to be accomplished, and mobilize resources within the work environment toward achieving their goals.

#### Subtheme: Taking Ownership in Self-Reflection on Own Behavior

Self-reflection was labeled by the participants as a form of self-reflection through introspecting and assessing their own actions throughout teaching and learning activities to ascertain whether these were still aligned with their personal values and goals on teaching, as quoted:

When I pick up that students are not understanding the lessons I sit back and probe for alternatives, to see if there are better approaches that could make students understand. If they fail, I ask my colleagues, what can I do to make things right? (G2 P5)

The participants opened themselves up to peers’ feedback on their teaching, a typical self-leadership practice, as this demonstrates self-awareness and taking the initiative on the part of the nurse educators, which leads to identifying alternative teaching methods. Therefore, a conducive climate that encourages the formation of a community of practice amongst educators, wherein nurse educators can feel safe sharing reflections without fear of judgment from colleagues, must be created.

#### Subtheme: Serving as Role Models Through Self-Care

There was overwhelming agreement amongst the participants that engagement in self-leadership implied serving as role models to peers and students. This is accomplished through nurse educators being aware of their responsibility as ideal characters and focal points, which provides an affirming outlook that can be emulated by their peers and students:

Students emulate our behaviors, and this positive influence extends to their professional development. Through excellence, integrity and self-care we foster a supportive and ethical atmosphere that is emulated by students. (G1 P1)

The participants, moreover, perceived self-leadership as an activity that could contribute toward positive professional socialization amongst students when they observe effective interpersonal relationships and demonstration of caring attributes, as quoted:

As dedicated nurse educators we should work with the institution to eradicate some of the bad conduct by a few amongst us…there are those amongst us that do not adhere to dress code, use foul language, chew gum in front of students, come late for class, have negative attitude and sometimes smell of alcohol… (G4 P4)

The participants viewed self-leadership as being aware of the influence one has on peers’ and students’ professional development through their demeanors, modeling positive values, emotions, and behaviors, often learned through observation. Participants believed that being a role model also meant motivating students to learn by being credible, respectful, and trustworthy in their work.

#### Subtheme: Self-Development

The participants described self-leadership as a process that is driven by intrinsic motivation, which requires nurse educators to take initiative to upskill themselves without any expectation from the employer. The following was mentioned by a participant:

We are in a developing world where every aspect of life is dynamic. So, if we remain stagnant and not changing in terms of our education and skills, then we remain static and redundant. Of course, then we need to be attending those workshops and seminars to be knowledgeable on current issues… (G4 P1)

The younger generation belong to the 21st century. So, we definitely need to update ourselves with new innovative teaching methods. (G4 P3)

The participants understood self-leadership as taking responsibility of their development by taking initiative of identifying own learning or professional development needs, centered on their understanding of the contemporary and futuristic developments. This self-driven activity is based on the premise that nurse educators are lifelong learners who need to constantly keep their knowledge and skills up to date, so that nurse educators have the competence to teach, innovate, and be confident to introduce new perspectives during teaching and learning activities. Therefore, the educational institution needs to support nurse educators' pursuits in lifelong learning by investing in their attendance at ongoing nurse educator development initiatives.

### Theme 2: A Supportive Healthy Academic Work Environment

The theme *a supportive healthy academic work environment* explores key factors that participants recognized as essential for fostering nurse educators’ teaching, scholarship, and service expectations. These factors, facilitated through management and peer support, include mentoring, collaboration, meaningful recognition, resource availability, and leadership approaches. Additionally, participants perceived these elements as instrumental in enhancing self-leadership, contributing to their professional growth, and improving student outcomes and overall institutional success.

#### Subtheme: Mentoring

Participants anticipated the availability of mentoring within the work environment, viewing guidance from experienced nurse educators and leaders as a key driver of self-leadership. However, such support was not always readily accessible, as indicated by the participants:

We need to be mentored, …. I realize that we could benefit a lot as young nurse educators, currently we do not have that direct guidance. We would benefit a lot if we had mentors. (G1 P6)

At times all you need is to be supported by the seniors…. you know when I arrived here, I was fortunate that somebody held me by hand… (G3 P2)

Mentoring refers to structured guidance and knowledge-sharing facilitated by experienced educators who support and empower their colleagues in self-leadership. Participants highlighted the importance of accessible mentors who offer individualized professional development opportunities, helping educators navigate challenges and enhance their leadership capabilities. The participants regarded mentoring as a valuable tool for exchanging knowledge, advice, and expertise between mentors and mentees. Additionally, they expected mentors to be readily available to assist new educators in fostering a positive and meaningful work experience. When institutions actively support mentoring initiatives, mentees feel empowered in their roles, while mentors gain acknowledgment for their leadership in developing others.

#### Subtheme: Collaborating

The participants regarded mobilizing of resources and thoughts in teams as a form of practicing self-leadership that leads to a harmonious work environment. The participants expressed themselves as follows in this regard:

As a team we look out for each other and learn from others. Even those that are furthering their studies, form study groups…. Of late there is a trend of studying for Masters, it used to scare us, now we face it together. (G1 P7)

We believe that teamwork is very crucial in nursing education because without it you cannot succeed as an institution. Working as a team ensures that our students are always attended to. We easily take over our colleague's class when they are not there. (G2 P4)

Collaborating differs from mentoring in that it involves teamwork and peer-driven interactions that promote the development of self-leadership by participating in teamwork and shared responsibilities. The participants’ views demonstrate their understanding that collaborating within teams or disciplines was a self-leadership practice that brings about cohesiveness amongst nurse educators. Thus, academic settings benefit when they provide platforms that support teamwork and collaboration, as it stimulates individual and team reflection and focus on institutional goals, creativity, commitment, and work engagement. Such environments also encourage the expression of diverse viewpoints and promote a culture of learning from one another.

#### Subtheme: Meaningful Recognition

Nurse educators indicated that self-leadership is facilitated when institutional management recognizes their successful accomplishment of goals and tasks. More specifically, they benefit when management provides recognition and appreciation when they meet goals. They expected equal and fair treatment and provided with given supportive guidance for demanding activities. The participants commented as follows:

We are not looking for something tangible or palpable from our managers [leaders]. Simply saying “We recognize and appreciate your efforts; you are doing well” is adequate for us. (G3 P3)

Sometimes you feel let down by lack of management support. This can be very frustrating. It is as if you are being tested based on your qualifications: “because she has PhD, let us see how she will do this”. (G1 P1)

The participants’ views above indicate that nurse educators feel motivated to work in an educational institution that exudes a caring working environment through appreciation and words of affirmation. Therefore, participants described their managers as caring if they made efforts to understand individual nurse educators' tasks, provided the necessary feedback, and offered individualized support that could lead to improved performance.

#### Subtheme: Supportive Management Leadership Style

Nurse educators preferred their leaders to employ a participative leadership style, and they described it as supportive and one that could promote their self-leadership because it allows interactive decision-making, as quoted:

I would recommend a participatory style of leadership because everybody the institution is viewed as important, all involved in planning, and always informed of what is happening.

Everyone is given a chance to participate in meetings, and not only a handful will be taking decisions for the institution. (G3 P4)

Institutional leaders were also regarded as supportive leaders if they ensured availability of required teaching and learning resources:

I would appreciate it if we had the necessary state of the art equipment necessary for teaching the current breed of students. (G4 P1)

The participants’ views demonstrate the significance of the leadership role played by institutional managers in creating motivating work environments. Participants were motivated when they were provided with a platform for input on institutional decisions. Likewise, they felt supported if provided with the latest teaching and learning infrastructure, as these would make their work challenging, interesting, and motivate their performance.

## Discussion

The aim of this study was to explore and describe nurse educators’ understanding of self-leadership and how it can be facilitated to promote a healthy academic work environment. Drawing on data from focus group interviews, the study revealed two overarching themes: (1) *Self-leadership means nurse educators being supported to take their own initiatives,* and (2) *A supportive healthy academic work environment*. Each theme was further elaborated through four subthemes that reflect key practices such as goal setting, self-reflection, role modeling, self-development, mentoring, collaboration, recognition, and supportive leadership. The findings suggest that nurse educators perceive self-leadership not merely as an individual capacity but as one that is nurtured through institutional support, collegial engagement, and value-aligned environments. Participants emphasized the importance of being empowered to initiate actions, reflect on their own behaviors, and be acknowledged within a collaborative and respectful workplace culture.

These findings align with self-leadership theories, particularly self-regulation (Carver & Scheier, 1981), self-control (Thoresen & Mahoney, 1974), and self-management (Manz, 1983), which emphasize individuals’ internal regulation of thoughts, motivation, and behaviors. These theories highlight the role of goal setting, self-reflection, and self-development in enhancing personal and professional growth, foundational elements in fostering a healthy academic work environment.

Bandura's social cognitive theory (Bandura, 1977, 1986) explains self-leadership as an ongoing interaction between individuals and their environment, shaping their motivation and behaviors. This aligns with the study's findings, where nurse educators demonstrated self-leadership through self-reflection and role modeling while responding to institutional and peer influences, emphasizing the importance of a supportive and resourceful work environment.

Self-determination theory (Deci et al., 2017) emphasizes the role of autonomous motivation, where individuals engage in tasks with a sense of purpose and ownership. Nurse educators who feel supported in setting goals and developing professionally are more likely to persist in self-leadership behaviors, leading to greater job satisfaction and improved teaching effectiveness (Heier et al., 2024). A value-based institutional culture, as indicated by the study findings, fosters an environment where nurse educators align their vision, values, and goals with those of the institution, enhancing their self-leadership.

Transformational leadership theory (Daria & Gaytos, 2025) highlights the role of leaders in inspiring and facilitating self-leadership through vision, mentorship, and intellectual stimulation. The study's findings reflect this, as participants emphasized the need for mentoring, appreciation, and a supportive leadership style to sustain their self-leadership practices. Transformational leaders serve as role models, reinforcing behaviors that support self-reflection, professional development, and collaboration, ultimately contributing to a harmonious and empowering academic environment.

By integrating these frameworks, the study situates nurse educators’ self-leadership within broader theoretical constructs, emphasizing the importance of institutional support, leadership engagement, and intrinsic motivation in fostering a healthy academic work environment. The subthemes discussed below reflect the participants’ shared understanding of nurse educator self-leadership and their suggestions on how it can be facilitated in an educational setting to bring about a healthy work environment.

The results reveal that *Setting goals for the self towards a clear vision* is a powerful process through which nurse educators plan for their personal and professional future and motivate themselves to turn their vision into a reality. This is in line with DeRueda's (2024) assertion that self-leaders in educational environments articulate and promote a vision related to their professional practices. The results show that the nurse educators understood self-leadership as having a vision extending beyond the teaching environment. This awareness prompts the educators to change their mindsets and become futuristic in their thinking and planning (Herbst, 2022). This indicates that supportive academic work environments motivate nurse educators by creating a value-based culture that makes it easy for nurse educators to align their vision, values, and goals with those of the institution.

The study results reveal the need for nurse educators to take *ownership in self-reflection on own behavior.* This is an act of taking accountability that entails actively engaging in deep self-reflection and introspection with the aim of understanding own behaviors and taking action to improve such acts to contribute to efforts to turn around performance. This description is in line with George-Puskar et al.'s (2024) description of self-reflection, wherein the concept is identified as a self-leadership practice that gets described as an intrapersonal activity that comes into effect when an individual disengages from negative emotions aligned with preceding behaviors. The individual then reflects on the reactions of fellow educators and students triggered by the previous emotional state and subsequent behavior, then initiates a process of new goal setting and reassessing situations that could potentially give rise to such reactions (George-Puskar et al., 2024). Like Horton-Deutsch and Sherwood (2023) and Walker and Oldford (2020), this study found that the educational institution can support nurse educators’ engagement in self-reflection by providing a dedicated space for quiet reflection, meditation, and even reflecting in open dialog as a group.

In *Serving as role models through self-care*, the results reveal that nurse educators understand self-leadership as having role model attributes that inspire colleagues and students. This result aligns with Yahaya et al. (2021) assertion that individuals in similar positions as nurse educators manage their professional image through role modeling. The participants viewed effective communication as crucial in the teaching and learning milieu. Nurse educators contributed to a healthy academic work environment by being as proﬁcient in practicing role modeling communication skills as they were in their respective academic duties and skills (Boamah et al., 2021; Hamilton, 2021). In addition, effective communication and presentable physical appearance were adjudged to be some of the factors that preserve the image and credibility of the nursing profession (Godsey et al., 2020).

The results of this study concur with Bryan and Blackman (2019) and Matahela and Van Rensburg (2023b), noting that educational institutions can support nurse educators by championing self-care initiatives and practices. In fulfilling this responsibility, the institutions need to actively contribute to creating a healing environment that encourages deliberate practices aimed at enhancing self-care and overall well-being, as emphasized by Borges et al. (2022). In turn, nurse educators can face the inner life and know the “self” better, fostering resilience and work–life balance.

The subtheme *Self-development* reveals that although self-development is a self-directed initiative, the educational institution plays a vital role in providing educators with the necessary support that keeps them abreast with trends and innovations in the education sector and in their areas of expertise so that they can facilitate teaching and learning from an informed perspective. Embracing self-development emerges as a pivotal factor in cultivating healthy and healing work environments (Borges et al., 2022). In this regard, a study by Saunders et al. (2021) found that a healthy academic work environment supports nurse educators’ self-development initiatives by providing a continuous learning environment and allocating them to teach in their areas of didactic and clinical expertise. One of the self-development activities that nurse educators can consider is self-leadership training.

Educators can be trained in self-leadership agility to adjust high emotional intensity and impulsive behavior so that there are smooth personal and professional interactions (Hofert, 2022). The leadership training aimed at the educators provides an opportunity to learn how to lead in ways that facilitate nurse educators’ self-leadership (Hamilton, 2021). An academic environment that demonstrates a supportive climate provides nurse educator leadership development opportunities. In line with results from other subthemes discussed above, effective development opportunities facilitate nurse educators’ self-development attributes. These include ability to self-explore, ability to engage in caring and thoughtful interactions with others, possession of self-conﬁdence when facing challenges, and believing in own leadership potential to transform nursing education (Horton-Deutsch & Sherwood, 2023; Miles & Scott, 2019).

The results of this study reveal that nurse educators viewed *Mentoring* as an intervention that could encourage their self-leadership. While the participants expected a traditional mentor–mentee relationship type focused on experienced or senior educators guiding the newer educator, mentoring that is beneficial for both mentor and mentee in an educational setting is known as reciprocal mentoring (Peterson & Ramsay, 2021). Reciprocal mentoring allows for learning from different perspectives of sociodemographic backgrounds, thus fostering diversity, collaboration, and confidence. Goldsby et al. (2020) recommend that a mentor have strong self-leadership skills and emotional maturity to facilitate the mentee's self-leadership. Contrary to the expectation of the participants, there are times when mentors may not be available in academia (Nemeth et al., 2021). In such situations, nurse educators can accept responsibility for their personal and professional growth through identifying developmental needs, developing and maintaining their personal developmental plans, and mobilizing internal and external resources to realize the needs (Nemeth et al., 2021).

Another supportive intervention that was viewed as facilitative of self-leadership was *Collaborating*. The results reveal participants’ acknowledgment that there may be areas in which they may have limited knowledge and experience, thus accepting that they may have suboptimal resources required for executing their teaching and learning activities. In such a situation, Zurba et al. (2022) advise engagement in collaborative tasks or projects, wherein access to diverse expertise from supportive colleagues is guaranteed. Some of the collaborative tasks that support educator self-leadership include discourses on designing innovative curricula, methods and practices of teaching and learning, and assessment activities (Wrigley & Mosely, 2022). In line with participants’ expectations, Saunders et al. (2021) recommend that the work environment fosters collaboration aligned to its vision, values, and goals through the promotion of teamwork, development of mutual goals, interdisciplinary teaching, and integration of knowledge and practice.

The subtheme *Meaningful recognition*, in line with Eddy et al. (2021), Hollinger-Smith et al. (2021), and Saunders et al. (2021) was described as when the individual nurse educator is recognized or appreciated for their work in a timely fashion, commensurate with the level of effort of the individual nurse educator and aimed at promoting reflective practices. Thus, to meet the nurse educators’ expectations of working in a work environment that supports, appreciates, and recognizes their efforts, there should be awareness of the effort and value brought about by each nurse educator, with strategies to implement the recognition meaningfully. For example, appreciation could be in the form of “thank you” gestures, incentives such as pay raises, flexible working hours, improved fringe benefits, and opportunities to craft a resourced pathway for career growth (Seymour, 2022).

For *Supportive management leadership styles*, the results reveal that participants appreciated the pivotal role played by the designated leader's leadership style in providing the motivation required for the nurse educators’ attainment of improved performance to reach higher goals. In line with study findings from Saunders et al. (2021), participants preferred the participative style of leadership, describing it as one that could stimulate their self-leadership practices. Managers in such a work environment value and respect their employees and involve them in decision-making processes so that there is shared governance in the institution and its departments (Saunders et al., 2021). The workplace could ensure consistent motivation of its nurse educators when institutional leaders become supportive academic leaders who champion institutional vision by innovatively leveraging adequate resources to nurse educators even during times of adversity (Hamilton, 2021; Williamson et al., 2021).

### Strengths and Limitations of the Study

This study has several strengths. First, it provides a rich, in-depth exploration of nurse educators’ perspectives on self-leadership, a topic that has received limited attention in nursing education research. The use of focus group discussions allowed for dynamic interactions, enabling participants to reflect on and expand each other's insights, leading to a more nuanced understanding of self-leadership. Additionally, the study included nurse educators from diverse institutions—private nursing schools, public nursing colleges, and university-based nursing departments, ensuring a broad representation of experiences and professional contexts. The rigor of the qualitative data analysis process, including independent co-coding and adherence to Tesch's protocol, further strengthened the credibility and trustworthiness of the findings.

Despite these strengths, the study has some limitations. While this study achieved data saturation, one limitation is that it focused solely on nurse educators’ perspectives, without including the views of institutional leaders or policymakers who play a crucial role in shaping the policies and work environments within nursing education institutions. Their perspectives could have provided additional insights into the structural and policy-level factors influencing self-leadership in academic settings. Only female nurse educators participated in the study. This limitation is directly related to the low number of male nurse educators working in the academic environment. Additionally, the study was geographically limited to two provinces in South Africa, which may influence the transferability of the findings to other regions. However, the findings could still be relevant to similar institutions operating under comparable educational and healthcare systems.

### Implications for Practice

Individuals and institutions are equally responsible for creating healthy work environments through collective endeavors; institutions, nurse educators, and students, for all to flourish. Fostering healthy academic environments in nursing education is a shared responsibility among institutions, nurse educators, and students. This study underscores the importance of self-leadership in enhancing personal growth, resilience, and professional effectiveness.

To translate findings into practice, institutions should integrate self-leadership into development programs by including brief courses in orientation and mentorship, scheduling regular reflective practice sessions, and promoting informal peer dialogs such as brown bag discussions and healing circles. Performance development plans can also guide self-regulation and growth. To mitigate emotional fatigue, peer support mechanisms should be simple and sustainable. Initiatives such as peer-pairing or triads for mutual check-ins and brief emotional reflections during departmental meetings can foster support. Piloting these interventions before scaling is recommended.

Given time constraints, nurse educators may benefit from microlearning workshops (30–60 min) focused on targeted topics such as mindfulness or goal setting. Offering continuing professional development points and incentives can enhance engagement, while partnerships with professional associations may support delivery and funding. For institutional endorsement, pilot data (e.g., satisfaction surveys, reflection logs) can support a one-page policy brief. A dedicated task team should codevelop a self-leadership support guideline, ensuring relevance and feasibility. To overcome implementation barriers, departments should identify leadership champions, run a six-month pilot with minimal resources, and train internal facilitators to lead reflective practices.

Evaluating these initiatives requires clear indicators, such as educator satisfaction, teaching effectiveness, innovation, retention, and team collaboration. Data can be collected through self-leadership scales, reflections, institutional records, and focus groups. These efforts can contribute to a more empowered, collaborative, and resilient nursing education workforce.

## Conclusions

This exploratory and descriptive qualitative study provided insights into nurse educators’ understanding of self-leadership and how it can be facilitated within educational institutions. The findings highlight the transformative potential of self-leadership in fostering healthy, resilient, and engaged academic environments. By integrating self-leadership principles into nursing education, practice, and policy, institutions can empower nurse educators to take ownership of their professional growth, enhance their engagement, and contribute meaningfully to their work environments and communities.

Beyond academia, the implications of this study extend into healthcare systems and policy development, reinforcing the need for leadership models that prioritize well-being, autonomy, and collaboration. Self-leadership not only strengthens nurse educators’ professional agency but also serves as a catalyst for creating sustainable, supportive workplaces that enhance both educator and student experiences. In turn, this cultivates a ripple effect, shaping healthcare environments that prioritize holistic well-being, compassionate leadership, and systemic resilience.
